# Depression and Anxiety Symptoms Are Associated with Mean Platelet Volume in Autoimmune Disorders

**DOI:** 10.3390/ijerph191711006

**Published:** 2022-09-02

**Authors:** Balázs Fábián, Ildiko Fanny Horváth, Amir Houshang Shemirani, Zoltán Csiki

**Affiliations:** 1 Department of Behavioural Sciences, Faculty of Medicine, University of Debrecen, 4032 Debrecen, Hungary; 2 Division of Clinical Immunology, Faculty of Medicine, University of Debrecen, 4032 Debrecen, Hungary; 3 Department of Laboratory Medicine, Division of Clinical Laboratory Sciences, Faculty of Medicine, University of Debrecen, 4032 Debrecen, Hungary

**Keywords:** mean platelet volume, anxiety, depression, platelet distribution width

## Abstract

Platelets are increasingly considered a bridge between mental and immunological disorders. However, data relating to platelet parameters in patients with autoimmune disorders are limited. The aim of the present study was to investigate, for the first time, the association of platelet parameters with the symptoms of affective disorders in patients with autoimmune conditions. In this cross-sectional study, we measured the complete blood count (CBC), the Generalized Anxiety Disorder Scale for anxiety (GAD-7), and the Beck Depression Inventory for depression (BDI) in 121 patients with autoimmune disorders. Mean platelet volume (MPV) was positively correlated with both anxiety and depression. Platelet distribution width (PDW) was negatively correlated with anxiety and depression. Before adjustment for covariates, logistic regression analysis revealed a significant association of MPV with depression and anxiety. After adjustment for covariates, only depression was associated with MPV. The area under the ROC curve of MPV for GAD-7 determined anxiety and BDI determined depression was 0.63. Our study showed that among the CBC hematological parameters, the MPV might be a useful biomarker of depression and anxiety in patients with autoimmune disorders. Further investigations of platelet parameters in controlled prospective studies are warranted to confirm our preliminary results.

## 1. Introduction

Anxiety and depression are the two most common pervasive psychiatric disorders in patients with chronic illnesses. Several autoimmune diseases have been shown to be associated with higher rates of anxiety [1,2] and depression [3,4]. The high comorbidity of these conditions raises the question of whether they evolve as a specific result of a pathology-inducing constellation of biological, psychological, and environmental factors. It is possible that a particular environmental trigger (e.g., stress) may instigate biological processes favoring the simultaneous development of somatic and mental disorders, where the biological mediating factors could serve as a biomarker of subsequent illness. Even though multiple mechanisms have been proposed to explain the relationship between affective disorders and somatic conditions, the exact underlying physio-pathological mechanisms remain poorly understood. Recent evidence suggests that dysregulated inflammatory responses are involved in the pathophysiology of a broad range of affective disorders including major depression disorder, bipolar disorder, and anxiety disorders [5,6,7,8,9,10].

Evidence supported by several studies indicates that changes in platelet parameters such as distribution width (PDW), and mean platelet volume (MPV) may arise from an underlying systemic inflammation [11,12,13].

Since platelets represent the greatest location for the storage of serotonin—one of the key neurotransmitters in the pathophysiology of depression—and share several features with neurons, several studies have investigated the relationship between affective disorders and platelet parameters [14]. Increased MPV was closely linked with anxiety and depressive disorders in cardiovascular diseases such as myocardial infarction or ischemic heart diseases [15,16]. Higher MPV was found compared to the control group in patients with panic disorder [17,18], depressive disorder [19,20], and anxiety disorder [19]. Increased PDW was found in panic disorder [21,22] and in patients affected by recurrent depression resistant to treatment [23]. In a large Italian population cohort study (N = 12,732), a significant positive association between depressive symptoms and PDW was found, even after adjustment for several socio-demographic characteristics [24].

In this study, we aimed to investigate whether anxiety and depression are associated with MPV and PDW in outpatients with autoimmune disorders. Additionally, we aimed to explore the predicting value of these biomarkers for anxiety and depression symptoms.

## 2. Materials and Methods

### 2.1. Participants and Procedure

Patients were recruited via the Outpatient Clinic of the Department of Internal Medicine, University of Debrecen. The inclusion criteria were: (a) the ability to understand and write Hungarian and (b) a definite diagnosis of an autoimmune disorder. Exclusion criteria were the presence of hematological disorders, mental retardation, autistic disorder, organic brain damage, psychotic symptoms, infections during the last 14 days, alcohol and/or drug abuse, current pregnancy, and current use of antipsychotic or antidepressant medications. Data were obtained by questionnaires, and sampling of blood. The study was approved by the University of Debrecen Clinical Centre’s Regional and Institutional Ethics Committee and the Scientific and Research Ethics Committee. Written informed consent was obtained from all participants. The study was conducted according to the Declaration of Helsinki.

### 2.2. Measures

Demographic data (age, gender, education, and family status), vascular risk factors (congestive heart failure, coronary heart disease, history of stroke, hypertension, diabetes mellitus), and medical history were obtained at baseline.

The short version of the Beck Depression Inventory (BDI) was used for assessing the symptoms of depression. Each item represents a single symptom associated with depression including sadness, crying, feelings of hopelessness, feelings of guilt, and sleep disturbance over the past two weeks. This questionnaire contains nine items each assessed on a scale from 0 (lack of depressive symptoms) to 3 (severe depressive symptoms). The total score may be categorized into four severity groups after conversion: minimal or no depression (0–13), mild (14–19), moderate (20–28), and severe (29–63). Twenty points had been determined as the optimal cut-off value for BDI [25].

To measure anxiety, the Generalized Anxiety Disorder Scale (GAD-7) was used. This questionnaire also contains seven items, and each is assessed on a scale of 0 (lack of anxiety symptoms) to 3 (severe anxiety symptoms). Thus, the total score of the questionnaire ranges from 0 to 21. Scores are classified in the following way: minimal or no anxiety (0–4), mild (5–9), moderate (10–14), and severe anxiety (15–21). Ten points were assessed as the optimal cut-off value for GAD-7 [26,27].

We measured hematological parameters from complete blood count. Blood samples were drawn from each subject in the morning after a fasting period of 12 h. The blood samples were collected into tubes containing EDTA and analyzed within two hours by the Sysmex SF-3000 analyzer (Sysmex Corporation, Kobe, Japan). All measurements were performed on the same instruments throughout the study period.

### 2.3. Statistical Analysis

Statistical analyses were carried out with IBM SPSS version 23.0 for Windows (SPSS, Inc., Chicago, IL, USA). The data were presented as the mean ± SD values or percentages as appropriate. The normality of data was evaluated using the Kolmogorov–Smirnov test. Continuous variables between anxiety and depression subgroups were compared by using the Mann-Whitney U test. The relationships between continuous variables were tested using Pearson’s correlation analysis. Anxiety and depression scores were divided into categories according to their severity, and participants in the first group (no or minimal anxiety or depression) were considered as a reference group. Scales measuring anxiety and depression were dichotomized using cutoff points suggested in the literature. Scores of 20 or greater on the BDI were considered indicative of elevated depression symptoms, and scores of 10 or greater on the GAD-7 defined elevated anxiety symptoms. To further evaluate the association of depression and anxiety symptoms with immunological measures, odds ratios (OR) with 95% confidence intervals (CI) were calculated using logistic regression. Receiver operating characteristic (ROC) curve analysis was used to evaluate the diagnostic value of platelet parameters. All the analyses were two-sided and a *p*-value of <0.05 was considered significant. The proportion of missing data was below 5% for each variable. Missing data were replaced by the respective sample mean.

## 3. Results

A total of 121 patients (mean age 55.47 ± 13.36 years) with autoimmune disorders were included in the study. Among them, 34 patients (28.1%) had at least moderate depression, and 35 patients (28.9%) had at least moderate anxiety. The socio-demographic characteristics and clinical and laboratory parameters of the study population were reported in Table 1.

First, CBC parameters were compared across groups determined by cut-off scores of the BDI and GAD-7 depression and anxiety scales. MPV was significantly higher (*p* = 0.028; *p* = 0.031) in those study subgroups where BDI and GAD-7 scores indicated at least moderate symptoms, respectively. HTC was significantly lower (*p* = 0.029) in the group where patients reported at least moderate anxiety (see Table 2). Overall, the CBC parameter means were in the normal range.

MPV was positively correlated with anxiety (r: 0.317, *p* < 0.01) and depression (r: 0.376, *p* < 0.01), while PDW was negatively correlated with anxiety (r: –0.245, *p* < 0.01) and depression (r: −0.238, *p* < 0.01) (see Table 3). There was a significant negative association between MPV and PDW (r: −0.374, *p* < 0.01).

Unadjusted and adjusted logistic regression analysis for BDI determined depression and GAD-7 determined anxiety using the hematological parameters in study participants are presented in Table 4 and Table 5, respectively. In the first step, every hematological parameter was entered in the logistic regression analysis simultaneously without adjustment for any potentially confounding factor. MPV showed a significant association with depression (OR = 1.720, *p* = 0.012) and anxiety (OR = 1.608, *p* = 0.031). To control the effects of potential confounding factors, the regression analyses were repeated by controlling for age, gender, education level, family status, diabetes, hypertension, and heart related disorders. After adjustments, MPV remained significantly associated with depression (OR = 2.003, *p* = 0.012), while its association with anxiety was not significant.

We performed receiver operating characteristic curve analyses to compare the ability of each parameter to predict the diagnostic categories based on the cut-off scores of the self-report measures of depression and anxiety. The area under the curve (AUC) for the MPV was 0.63 for predicting both depression (*p* = 0.028) and anxiety (*p* = 0.031). The optimal cut-off value for the MPV was 7.84 fL, and its sensitivity and specificity for anxiety were 63% and 59.3%, respectively. The optimal value for the MPV was 7.99 fL for depression with 53% sensitivity and 67.8% specificity. For other parameters, including PDW, there were no significant results.

## 4. Discussion

This study is the first to evaluate symptoms of affective disorders and hematologic indices in patients with autoimmune disorders. In the last few years, studies have increasingly focused on identifying objective makers that can be used for clarifying the etiology of psychiatric diseases, risk detection, diagnosis, prediction, and developing treatment strategies [28]. Because of this, several studies have recently been conducted on platelets and platelet indices, such as MPV, PDW, or red cell distribution width (RDW) in psychiatric populations. Measuring these parameters is simple and relatively inexpensive, which makes them good candidates for frequent assessment. Neurons and platelets have similar functional and structural features. Thus, platelets are often utilized as peripheral models of central neural activity and biochemical changes, especially serotonin metabolism [29,30,31,32,33].

MPV has been suggested as a useful biomarker in psychiatric conditions [34,35,36]. It has been reported that MPV was found to be elevated in patients with major depression (N = 15) when compared with healthy individuals [37]. In a larger non-clinical sample, the authors replicated the same results: depressed individuals exhibited increased platelet activation in comparison with individuals without major depression [38]. Among chronically stressed caregivers, increased levels of depressive symptoms and anxiety are associated with greater platelet responsiveness and prolonged platelet activation [39]. The association between the MPV and panic disorder has been also investigated, however, these studies have shown limited and inconclusive findings [15,17,18,22]. The reasons for these contradictory results are unclear. Concordant with the previous findings, using correlation and regression analyses, we found increased MPV in patients with anxiety symptoms without adjustments, and with depression symptoms with and without adjustments for covariates. Elevated MPV values have been attributed to emotional stress [40], increased sympathetic activity in patients with depression [17,38,41,42], and in patients with generalized anxiety disorder [35]. More recently, changes in platelet parameters have been linked to inflammatory processes, thus an abnormal MPV might indicate inflammation in psychiatric conditions [43,44]. However, the exact mechanism behind the association of changes in platelet parameters and affective disorders is not yet clearly established, since there have been results showing significantly decreased MPV in major depression [45]. Further studies to evaluate platelet parameters and other inflammatory markers, such as interleukins, interferons, tumor necrosis factors, and especially immunoglobulins, in autoimmune disorders are warranted.

Even though PDW significantly correlated with BDI and GAD-7 scores, logistic regression analysis using PDW as a predictor variable did not demonstrate any significant association when covariates, such as MPV, were included in the analysis. ROC curve analysis indicated a non-significant predicting value for PDW. In previous studies, elevated PDW has been found in depression [24] and panic disorders [21,22] while other studies have found no such association in a panic [15] or generalized anxiety disorders [35]. Since we could not replicate these findings in our heterogeneous patient group, we could not recommend PDW as biomarkers for psychiatric conditions in autoimmune disorders. Further studies are required to clarify the connection between the changes in PDW and psychiatric symptomatology.

In two previous studies [20,44] it has been demonstrated that ROC curve analysis of PDW and RDW measurements is useful in differentiating healthy controls from the major depressive disorder and manic bipolar disorder groups. In a similar study [35] the suggested cut-off value for MPV was 7.45 fL, while its sensitivity and specificity for diagnosis of generalized anxiety disorder were 65% and 56.7%, respectively. The AUC was 0.66 in their study. Our present results regarding MPV are in line with previous findings [35], while in this study neither PDW nor RDW demonstrated significant predictive power over GAD-7 and BDI determined groups. ROC curve analysis in our study demonstrated an MPV cut-off value of 7.99 fL for BDI (*p* = 0.028) with a sensitivity of 53% and a specificity of 67.8%; and 7.84 fL for GAD-7 (*p* = 0.031) with a sensitivity of 63% and specificity of 59.3%. The AUC for the MPV was 0.63 for predicting both target groups. Since the sensitivity, specificity, and AUC values are quite low, we could not recommend using MPV as a diagnostic marker for depression or anxiety in general practice. However, ROC analysis confirmed the significant relationship between MPV and affective symptomatology found during regression analyses.

The results of the present study should be considered in the context of the following limitations. Firstly, even though current medication, body mass index, and serum lipid levels may affect platelet hematological parameters or inflammation, we could not control for these effects since the study was performed retrospectively. Secondly, the study sample was relatively small. Therefore, we could not perform group comparisons based on diagnosis or examine the effect of the co-occurrence of anxiety and depression. The overlap of patients with both at least moderate depression and anxiety was 20.7%. Third, we used only self-report measures for the assessment of anxiety and depression. The presence of psychiatric disorders was not supported by clinical interviews. Moreover, most of the study population (85.1%) was female; thus, our results may not be generalizable for both genders. The findings and their implications should be discussed in the broadest context possible. Future research directions may also be highlighted.

## 5. Conclusions

In conclusion, this study is the first to demonstrate that MPV is associated with the symptoms of depression and anxiety in a heterogenous sample of patients with autoimmune disorders. It seems that the increase in MPV might be attributed to the pathological activity of the inflammatory system. Since hematological inflammatory parameters are routinely reported, their measurement is inexpensive, simple to calculate, and they could be easily used in the first line of detecting psychiatric disorders. Based on our findings, we believe that medical professionals might improve overall treatment outcomes by using MPV as an initial indicator for psychiatric conditions and facilitating further psychological assessment among patients with autoimmune disorders. Nevertheless, further research involving the evaluation of the exact function of platelets and the importance of MPV as a tool for predicting affective disorders is required to confirm these findings. MPV alone should not be used as a measure of platelet activity, since MPV may be affected by cardiovascular diseases or other pathologies. The predicting power of the combination of different hematological parameters should be investigated in the future. Prospective clinical studies are required to investigate the effect of treatment on platelet hematological parameters.

## Figures and Tables

**Table 1:** Demographic and Clinical Characteristics of Participants.

Characteristics	
Demographics	
Gender (women) [N (%)]	103 (85.1)
Age (years) [mean (SD)]	55.47 (13.36)
Education (years) [mean (SD)]	12.72 (2.59)
Family status (single) [N (%)]	35 (28.9)
Diagnosis	
Sjögren’s syndrome [N (%)]	38 (31.4)
Rheumatoid arthritis [N (%)]	37 (30.6)
Primary Raynaud’s disease [N (%)]	29 (26.4)
Systemic lupus erythematosus [N (%)]	9 (7.4)
Systemic sclerosis [N (%)]	4 (3.3)
Vascular Risk Factors	
Hypertension [N (%)]	15 (12.4)
Diabetes mellitus [N (%)]	7 (5.8)
Congestive heart failure [N (%)]	5 (4.1)
Coronary heart disease [N (%)]	4 (3.3)
History of stroke [N (%)]	2 (1.7)
Depression (at least moderate) [N (%)]	34 (28.1)
Anxiety (at least moderate) [N (%)]	35 (28.9)
Laboratory Data	
MPV (fL) [mean (SD)]	8.07 (1.21)
PDW (fL) [mean (SD)]	51.78 (13.52)
HTC (%) [mean (SD)]	41.3 (2.99)
WBC (109/L) [mean (SD)]	6.96 (1.94)
RBC (1012/L) [mean (SD)]	4.73 (0.37)
HGB (g/dl) [mean (SD)]	13.41 (2.22)
MCV (fL) [mean (SD)]	86.77 (9.09)
RDW (%) [mean (SD)]	13.46 (0.95)

Note: MPV = mean platelet volume; PDW = platelet distribution width; HTC = hematocrit; WBC = White blood cell; RBC = red blood cell; HGB = haemoglobin; MCV = mean corpuscular volume; RDW = red cell distribution width.

**Table 2:** Complete blood parameters in the study groups. Variables are summarised as mean (standard deviation).

	No Depression	Depression	No Anxiety	Anxiety
MPV	7.85 (0.9)	8.63 (1.66) *	7.88 (0.99)	8.52 (1.54) *
PDW	53.06 (10.53)	48.51 (18.97)	53.45 (10.89)	47.70 (18.01)
HTC	41.28 (3.018)	41.35 (2.94)	41.59 (2.97)	40.57 (2.94) *
WBC	6.98 (2.06)	6.99 (1.6)	6.90 (1.88)	7.11 (2.09)
RBC	4.76 (0.37)	4.67 (0.33)	4.76 (0.37)	4.66 (0.32)
HGB	134.06 (21.66)	134.34 (23.68)	133.49 (25.36)	135.74 (10.96)
MCV	86.96 (5.69)	86.28 (14.69)	87.49 (5.73)	85.01 (14.32)
RDW	13.52 (0.96)	13.31 (0.9)	13.47 (0.99)	13.45 (0.84)

Note: MPV = mean platelet volume; PDW = platelet distribution width; RDW = red cell distribution width; HTC = hematocrit; WBC = White blood cell; RBC = red blood cell; HGB = haemoglobin; MCV = mean corpuscular volume. * *p* < 0.05.

**Table 3:** Correlations between hematological parameters with anxiety and depression symptoms.

	MPV	PDW	HTC	WBC	RBC	HGB	MCV	RDW
Anxiety	0.32 **	−0.25 **	−0.16	0.07	−0.16	−0.03	−0.15	−0.05
Depression	0.38 **	−0.24 **	−0.01	0.01	−0.14	0.03	−0.08	−0.1

Note: MPV = mean platelet volume; PDW = platelet distribution width; RDW = red cell distribution width; HTC = hematocrit; WBC = White blood cell; RBC = red blood cell; HGB = haemoglobin; MCV = mean corpuscular volume. ** *p* < 0.01.

**Table 4:** Logistic regression analysis for BDI determined depression.

	Unadjusted			Adjusted ^a^		
	Odd Ratios	95% CI	*p* Value	Odd Ratios	95% CI	*p* Value
MPV	1.720	1.129–2.619	0.012 *	2.003	1.168–3.434	0.012 *
PDW	0.994	0.960–1.028	0.715	0.987	0.947–1.029	0.545
HTC	1.132	0.775–1.654	0.522	1.155	0.322–4.144	0.826
WBC	0.942	0.733–1.212	0.644	0.900	0.645–1.254	0.532
RBC	0.253	0.009–7.467	0.426	0.108	0.001–34.635	0.699
HGB	1.002	0.982–1.023	0.823	1.002	0.976–1.028	0.893
MCV	0.930	0.794–1.089	0.367	0.875	0.466–1.643	0.679
RDW	0.684	0.383–1.222	0.200	0.637	0.304–1.337	0.234

Note: MPV = mean platelet volume; PDW = platelet distribution width; HTC = hematocrit; WBC = White blood cell; RBC = red blood cell; HGB = hemoglobin; MCV = mean corpuscular volume; RDW = red cell distribution width. ^a^ Adjusted for age, gender, education level, family status, diabetes, hypertension, congestive heart failure, coronary heart disease, and history of stroke. * *p* < 0.05.

**Table 5:** Logistic regression analysis for GAD-7 determined anxiety.

	Unadjusted			Adjusted ^a^		
	Odd Ratios	95% CI	*p* Value	Odd Ratios	95% CI	*p* Value
MPV	1.608	1.044–2.477	0.031 *	1.642	0.978–2.758	0.061
PDW	0.986	0.951–1.023	0.445	0.974	0.932–1.019	0.252
HTC	0.774	0.384–1.562	0.474	0.805	0.250–2.597	0.717
WBC	0.997	0.791–1.256	0.979	1.041	0.786–1.379	0.779
RBC	0.365	0.002–74.407	0.711	0.043	0.001–72.89	0.526
HGB	1.078	0.959–1.212	0.206	1.137	0.981–1.319	0.089
MCV	0.912	0.678–1.227	0.544	0.792	0.446–1.404	0.424
RDW	0.900	0.503–1.612	0.724	1.019	0.500–2.076	0.958

Note: MPV = mean platelet volume; PDW = platelet distribution width; HTC = hematocrit; WBC = White blood cell; RBC = red blood cell; HGB = hemoglobin; MCV = mean corpuscular volume; RDW = red cell distribution width. ^a^ Adjusted for age, gender, education level, family status, diabetes, hypertension, congestive heart failure, coronary heart disease, and history of stroke. * *p* < 0.05.

## Data Availability

The entire dataset cannot be made publicly available since the analysis is still in progress. After the completion of the ongoing work, excerpts of the data can be obtained from the first authors upon reasonable request.
